# Measurement of ^18^O^18^O and ^17^O^18^O in atmospheric O_2_ using the 253 Ultra mass spectrometer and applications to stratospheric and tropospheric air samples

**DOI:** 10.1002/rcm.8434

**Published:** 2019-05-08

**Authors:** Amzad H. Laskar, Rahul Peethambaran, Getachew A. Adnew, Thomas Röckmann

**Affiliations:** ^1^ Institute for Marine and Atmospheric Research Utrecht Utrecht University The Netherlands

## Abstract

**Rationale:**

The doubly substituted isotopologues (e.g., ^18^O^18^O, ^17^O^18^O) in atmospheric O_2_ are potential tracers for ozone photochemistry and atmospheric temperatures. Their low abundances and isobaric interference are the major analytical challenges. The 253 Ultra high‐resolution stable isotope ratio mass spectrometer is suitable for resolving isobaric interferences.

**Methods:**

O_2_ from air is purified using gas chromatography on a packed column filled with molecular sieve 5 Å and cooled to −78°C. The δ^17^O, δ^18^O, Δ^17^O, Δ_35_ and Δ_36_ values are measured on the extracted O_2_ with the 253 Ultra at medium mass resolution (M/ΔM ~10000) using Faraday detectors for the singly substituted isotopologues and ion counters for the doubly substituted isotopologues.

**Results:**

Interferences from isobars, mainly ^35^Cl for ^17^O^18^O and H^35^Cl and ^36^Ar for ^18^O^18^O, are sufficiently resolved to enable high‐precision determination of Δ_35_ and Δ_36_. The Δ_35_ and Δ_36_ values of O_2_ after photochemical isotope equilibration at −63°C and heating to 850°C agree with the theoretical prediction. The stratospheric Δ_35_ and Δ_36_ values are close to isotopic equilibrium at the ambient temperatures. However, the values for tropospheric O_2_ differ from those expected at equilibrium.

**Conclusions:**

The 253 Ultra allows interference‐free clumped isotope measurements of O_2_ at medium mass resolution. The Δ_35_ and Δ_36_ signatures in atmospheric O_2_ are mainly governed by O_3_ photochemistry, temperature and atmospheric transport. Tropospheric O_2_ is isotopically well mixed and retains a significant stratospheric signature.

## 1 INTRODUCTION

Molecular oxygen (O_2_) is an important constituent of the Earth's atmosphere and it is intricately linked to most life forms on earth. The atmospheric O_2_ reservoir exchanges with the biosphere on a timescale of roughly 1200 years.^1^ Its isotopic composition is affected by biological, hydrological and photochemical processes.^1^, ^2^, ^3^, ^4^, ^5^, ^6^, ^7^ However, only the bulk isotopic ratios of O_2_ (i.e., ^18^O/^16^O and ^17^O/^16^O) have been studied extensively. In nature, these ratios vary primarily in response to biological oxygen cycling, although the influence of stratospheric photochemistry has also been observed.^2^, ^6^, ^7^, ^8^, ^9^, ^10^ The doubly substituted isotopologues, ^18^O^18^O, ^17^O^18^O and potentially also ^17^O^17^O, also called clumped isotopes, can be useful to independently constrain the cycling of oxygen in the Earth's atmosphere.^7^

Clumped isotope signatures in O_2_, expressed as Δ_35_ and Δ_36_ (see definitions below), are quantified as deviations of the abundances of ^17^O^18^O and ^18^O^18^O, respectively, from the abundances that are expected stochastically from the bulk isotopic composition. Under thermodynamic equilibrium conditions, the magnitude of the deviations purely depends on the temperature at which isotopic equilibration of O_2_ takes place. The main challenges to measuring clumped isotope signatures in atmospheric O_2_ are their low abundances (1.6 ppm for ^17^O^18^O and 4 ppm for ^18^O^18^O) and the isobaric interferences, for example, the interference of ^36^Ar on ^18^O^18^O. Using careful separation and appropriate corrections for this interference, clumped isotope compositions in O_2_ were first measured with low‐resolution mass spectrometers.^6^, ^11^ Yeung et al^7^ later showed with a medium‐resolution isotope ratio mass spectrometer (modified Nu Perspective IS, CAMECA, Gennevilliers, France, mass resolving power ~3600) that separation of ^18^O^18^O from ^36^Ar is possible. Here we demonstrate that the 253 Ultra high‐resolution stable isotope ratio mass spectrometer (Thermo Fisher Scientific, Bremen, Germany) can measure the Δ_35_ and Δ_36_ values along with the traditional isotope ratios (δ^17^O and δ^18^O values) at a precision close to the counting statistics limit. We show that traces of ^36^Ar and other interferences can be well separated from ^17^O^18^O and ^18^O^18^O at medium mass resolution (~10000).

The first clumped isotope studies showed that Δ_35_ and Δ_36_ values are mainly governed by O_2_ + O(^3^ *P*) photochemistry in the atmosphere.^6^, ^7^, ^11^ Significantly higher values of Δ_35_ and Δ_36_ in the Earth's atmosphere than expected from a stochastic distribution of isotopes led Yeung et al^6^ to hypothesize that O(^3^ *P*) + O_2_ isotope exchange reactions reorder the isotopes in O_2_ toward an isotopic equilibrium that depends on the ambient temperature. At lower temperatures, the ^17^O^18^O and ^18^O^18^O clumping should be relatively high, i.e., higher values of Δ_36_ and Δ_35_, and vice versa.^12^ Therefore, in thermodynamic equilibrium the clumped isotope signatures of O_2_ should vary throughout the atmosphere because of variations in temperature. Yeung et al^6^ observed that the clumped isotopes of stratospheric O_2_, above 22 km, are indeed in thermodynamic equilibrium at the low ambient stratospheric temperatures. In contrast, there was a clear deviation from isotopic equilibrium at the higher temperatures in the troposphere. This was explained in terms of the time scales of isotopic re‐equilibration, which are long in the troposphere because of a low abundance of O atoms and short in the stratosphere because of much higher O atom levels there. Thus, in different regions of the atmosphere the isotopic re‐equilibration can be faster or slower than the transport timescales. Therefore, the atmospheric Δ_35_ and Δ_36_ values reflect a dynamic balance between isotope exchange (temperature and oxidant dependent) and transport. Interestingly, the biological recycling of O_2_, which is of primary importance for its bulk isotopic composition, has a negligible effect on the clumped isotopic composition because of the slow resetting time scale compared with photochemical isotope exchange.^1^, ^7^

Here we report a method for measuring clumped and bulk stable isotope ratios in atmospheric O_2_ using the 253 Ultra mass spectrometer. After establishing and validating the purification and the mass spectrometric methodology, an empirical transfer function was developed to convert the measured isotope compositions into the absolute reference frame (ARF) by isotopically equilibrating O_2_ at low and high temperatures. We also report Δ_35_ and Δ_36_ values measured in stratospheric and tropospheric air O_2_ samples. In addition, we discuss the challenges and possibility of measuring the rarest isotopologue, ^17^O^17^O, of O_2_ using the 253 Ultra.

## 2 CLUMPED ISOTOPES IN O_2_ AND THEIR MEASUREMENTS

### 2.1 Conventional and clumped isotopes in O_2_

The conventional singly substituted isotopic composition of a gas is characterized by the delta value, i.e., δ^17^O = (^17^R_sam_/^17^R_std_ – 1) and δ^18^O = (^18^R_sam_/^18^R_std_ – 1), where ^17^R = ^17^O/^16^O, ^18^R = ^18^O/^16^O, and subscripts ‘sam’ and ‘std’ stand for sample and standard, respectively. The δ values are expressed in per mill (‰) or parts per thousand. Values of δ^17^O and δ^18^O are reported with respect to Vienna‐Standard Mean Ocean Water (V‐SMOW).^13^

The doubly substituted isotopologues of O_2_, also called clumped isotopes, are ^18^O^18^O, ^17^O^18^O and ^17^O^17^O. Their excess abundances compared with a stochastic distribution of the three oxygen isotopes in O_2_ (i.e., the isotopes are randomly distributed over all isotopologues) are expressed using the relationship Δ_*i*_ = (^*i*^*R*_measured_/^*i*^R_random_ − 1), where *i* refers to mass 36, 35 and 34 (e.g., ^36^ *R*_measured_ = [^18^O^18^O]_measured_/[^16^O^16^O]_measured_) and

$$\begin{array}{l} {}^{36}R_{\text{random}}=\frac{{\left[{}^{18}O{}^{18}O\right]}_{\text{random}}}{{\left[{}^{16}O{}^{16}O\right]}_{\text{random}}}=\frac{\left[{}^{18}O\right]\left[{}^{18}O\right]}{\left[{}^{16}O\right]\left[{}^{16}O\right]}={\left({}^{18}R\right)}^{2},\\ {}^{35}R_{\text{random}}=\frac{{\left[{}^{18}O{}^{17}O\right]}_{\text{random}}}{{\left[{}^{16}O{}^{16}O\right]}_{\text{random}}}=2\frac{\left[{}^{18}O\right]\left[{}^{17}O\right]}{\left[{}^{16}O\right]\left[{}^{16}O\right]}=2^{18}R^{17}R,\;\text{and}\\ {}^{34}R_{\text{random}}=\frac{{\left[{}^{17}O{}^{17}O\right]}_{\text{random}}}{{\left[{}^{16}O{}^{16}O\right]}_{\text{random}}}=\frac{\left[{}^{17}O\right]\left[{}^{17}O\right]}{\left[{}^{16}O\right]\left[{}^{16}O\right]}={\left({}^{17}R\right)}^{2} \end{array}$$

Δ_*i*_ values are also reported in ‰. For a stochastic distribution of isotopes, Δ_*i*_ = 0. A nonzero Δ_*i*_ value for a sample represents an excess or deficit of multiply substituted isotopes (e.g., ^18^O^18^O) compared with what is expected based on their bulk isotopic composition. We calculate the clumped isotope ratios following the previously adopted established conventions and data reduction procedures for O_2_ (Δ_35_ and Δ_36_) and CO_2_ (Δ_47_)^11^, ^14^, ^15^ (see supporting information for detailed calculation).

### 2.2 Measurement of O_2_ clumped isotopes with the 253 Ultra

Isotopic measurements including the clumped isotopes (Δ_35_ and Δ_36_) were carried out with the 253 Ultra isotope ratio mass spectrometer at Utrecht University, which is one of the first instruments produced by Thermo Fisher Scientific on the basis of the prototype described in Eiler et al.^16^ It is a double‐focusing mass spectrometer with an electrostatic analyzer followed by a magnetic sector. It can be operated at three different mass resolutions, set by one of the three slits between the source and the electrostatic analyzer. The widths of the slits are 250, 16 and 5 μm for low, medium and high mass resolutions, respectively. Mass resolving power (M/ΔM) is defined as the ratio between the nominal mass and the width, measured in mass units, that corresponds to an intensity increase from 5% to 95% of the maximum peak signal. The maximum mass resolving power achievable with the present instrument is ~40 000 with the narrowest slit. The typical mass resolving powers at low and medium resolution are ~2000 and ~10 000, respectively. The collector assembly has nine different collector positions, where the central collector is fixed and the other eight are movable. The central collector can be switched between a Faraday collector and a Secondary Electron Multiplier (SEM), and the three collector blocks at the high mass end contain both a Faraday collector and a Compact Discrete Dynode (CDD) SEM next to each other. The Faraday collectors can be operated using a choice of resistors from 3*10^8^ Ω to 10^13^ Ω. Operation of the instrument is controlled by the Qtegra software package (Thermo Fisher Scientific).

In the present work, the medium‐resolution slit with a mass resolving power of ~10 000 (M/ΔM) for O_2_ is used. The major isotopologues (^16^O^16^O, ^16^O^17^O, ^16^O^18^O) are measured in Faraday cups with resistors 10^10^ Ω, 10^12^ Ω and 10^11^ Ω, respectively, while the minor ion beams (^17^O^18^O, ^18^O^18^O) are measured using two CDDs.

Isotope ratio measurements are generally made at a source pressure of about 2.0 × 10^−7^ mb. Under these conditions, in medium‐resolution mode the ion signal is 1 × 10^10^ counts per second (cps) at mass 32; the corresponding signals for masses 35 and 36 are ~20 × 10^3^ and ~50 × 10^3^ cps, respectively. A sample is measured for 30 to 60 sample‐reference cycles, divided into 5 to 10 acquisitions, each with 6 cycles. Peak centering, background scanning (60 s) and pressure‐balancing (tolerance 0.3%) are performed before each acquisition. The equilibration time (idle time) and integration time are set as 60 s and 67 s, respectively. Measurement of a sample takes ~7 h which consists of an actual measurement time of ~4.5 h, roughly half of which is required for equilibration after valve switching and the other half for signal acquisitions of the sample and the working gas. The remaining ~2.5 h is used for background scanning, reference and sample signal balancing, peak centering and peak positioning (all of these are performed at the beginning of each acquisition).

### 2.3 Preparation of equilibrated gases for clumped isotope calibration

We isotopically equilibrate aliquots of O_2_ at different temperatures in order to express our measured clumped isotope ratios against a scrambled reference. At high temperatures (850°C), isotopic equilibration of O_2_ is carried out in the presence of a platinum catalyst (~120 mg of Pt mesh; Goodfellow, Huntingdon, UK) in a quartz tube of length 42 cm and diameter 1.5 cm. Heating is carried out by inserting ~30 cm of the tube into a furnace set at 850°C. After 2–3 h of heating, the tube is directly connected to one of the bellows of the dual‐inlet system of the 253 Ultra to transfer hot O_2_ for measurement.

At low temperatures, it is not straightforward to equilibrate O_2_ isotopically as it does not readily exchange isotopes. Therefore, UV‐induced photochemical isotope exchange^11^ is used to equilibrate a number of O_2_ gases at lower temperatures. UV photolysis experiments are carried out in a spherical ~500‐mL volume Pyrex reactor (diameter 10 cm) fitted with a Suprasil finger (outer diameter 2 cm, length 10 cm; LSP Quartz, Wijchen, The Netherlands) at the center. The Suprasil finger transmits UV radiation (Pyrex is opaque to UV), and it is connected to the reactor using a KF clamp with a Teflon O ring. A Hg lamp (NTE 5 W.210; Radium, Wipperfürth, Germany) is introduced into the Suprasil finger as the UV source (major wavelengths: 184.9 and 253.6 nm).

The reactor is connected to a gas mixing system^17^ with a 6 mm ultra‐torr fitting and through a vacuum stopcock with a Teflon seal. The reaction vessel is first evacuated to a high vacuum of 1*10^−5^ mbar and then pure O_2_ working gas (IMAU O_2_; Linde Gas, Schiedam, The Netherlands) is introduced into the vessel to a pressure of 40–50 mbar. The flask is immersed in an ethanol or water bath and cooled to various temperatures between +26°C and −78°C. While the outside of the reactor is at the temperature of the cooling fluid, determination of the temperature of the gas inside the reactor is challenging, because the temperature of the Suprasil finger with the glowing UV lamp is much higher than the outside temperature of the reactor. For example, when the outer surface is at dry‐ice temperature (−77.8°C), the temperature inside the Suprasil finger is found to be 10 ± 5°C at steady state. The effective temperature of the gas inside the flask is calculated from the change in pressure using Gay‐Lussac's pressure law P_1_/T_1_ = P_2_/T_2_, where P_1_ is the pressure inside the chamber at room temperature (T_1_), i.e. before putting the chamber inside the bath and without UV lamp, and P_2_ and T_2_ are the pressure and temperature with the chamber inside the dry‐ice bath and the glowing UV lamp inside the finger after stabilization. For the lowest bath temperature of −77.8°C the effective gas temperature is −63 ± 5°C. This effective temperature is then used to assign the expected isotopic ordering of O_2_ inside the flask based on the theoretical calculations of Wang et al.^12^ After irradiation for 2–3 h, the equilibrated O_2_ samples are separated from the O_3_ formed during the UV treatment by passing the gas through a spiral tube immersed in liquid nitrogen. It is observed that 3–4% of the O_2_ inside the chamber is converted into O_3_ at steady state. Therefore, immediate separation of O_3_ is necessary to prevent further O_2_ formation from decomposition of O_3_, which would probably change the isotopic signatures. The isotopically equilibrated O_2_ samples are measured without any further purification.

All samples are measured against a working gas (IMAU O_2_) with nominal δ^17^O and δ^18^O values of 9.254‰ and 18.542‰, respectively, versus V‐SMOW, assigned by E. Barkan at the Institute of Earth Sciences, Hebrew University of Jerusalem (Jerusalem, Israel).

### 2.4 Sample O_2_ purification

**Figure 1:**
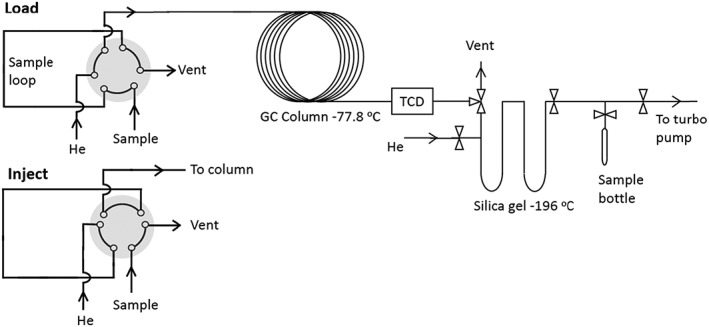
O_2_ purification system. An air sample or gas mixture is admitted to a sample loop (10‐mL volume) via a six‐port valve. O_2_ is separated from the other air constituents using a packed molecular sieve 5 Å (length: 3.05 m, OD: 1/8 inch, ID: 2 mm, Molsieve 5 Å) column cooled to dry‐ice temperature (−77.8°C). The effluent from the GC column is monitored using a thermal conductivity detector (TCD, see Figure 2) and the sample is collected on silica gel at liquid nitrogen temperature (−196°C)

**Figure 2:**
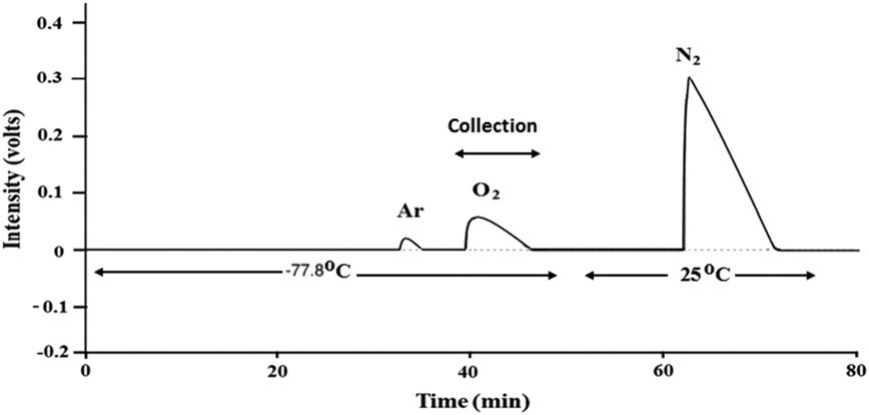
Gas chromatogram showing the separation of O_2_ from Ar and N_2_ in the GC column at dry‐ice temperature (−77.8°C). The separation between the Ar and O_2_ peaks is ~5 min when the GC column is kept at −77.8°C. Under these conditions N_2_ is trapped on the GC column. To release the N_2_ and observe the N_2_ peak on the chromatogram, the GC column was taken out of the dry ice/ethanol bath when O_2_ collection was finished, and kept at 25°C (indicated by the arrows below the detector trace)

O_2_ is separated from air using a gas chromatography (GC) column (column length: 3.05 m, OD: 1/8 inch, ID: 2 mm, Molsieve 5A; Agilent Technologies, Santa Clara, CA, USA). Air is filled into a sample loop of 10 mL volume (Figure 1) either by flushing air from a high‐pressure container or by using an air‐tight syringe of 50 mL capacity. The syringe is loaded with 50 mL of the air sample and is used to flush and fill the sample loop before injecting into the GC column. High‐purity He is used as the carrier gas at a flow rate of 25 mL/min. The GC column is kept at dry ice temperature (−77.8°C). The eluted gases are monitored using a Thermal Conductivity Detector (TCD). The Ar peak appears at ~32 min and O_2_ at ~40 min with a separation of ~5 min between the two peaks (Figure 2). We do not observe the N_2_ peak in the 80‐min time window set in the present case unless the column temperature is increased. To show the N_2_ peak (Figure 2), the column is removed from the dry‐ice bath and is kept at room temperature after O_2_ has eluted. The purified O_2_ sample is collected at liquid nitrogen temperature in two stainless steel U traps (OD: 6 mm) filled with silica gel. O_2_ is released from the U traps by removing the liquid nitrogen and then transferred to a glass ampule of ~2 mL volume filled with a few pellets of preconditioned silica gel. O_2_ from the ampule is directly introduced into the bellow of the mass spectrometer to measure its isotopic composition. After each sample extraction, the GC column is baked at 200°C for 1 h at a He flow rate of 25 mL/min and longer baking (overnight) is performed at the end of the day. The two silica gel U traps for sample collection are also baked at 200°C for 1 h under high vacuum (1*10^−5^ mb) after each sample collection.

### 2.5 Preparation of O_2_ for testing isotopic reordering

There is a possibility of isotopic reordering in O_2_ during sample storage, purification (e.g. GC column) and analysis in the mass spectrometer.^7^ To quantify potential reordering effects, we prepared enriched O_2_ (^18^O_2_ ~700‰) in a 2‐L volume canister by diluting 97.2 atom % ^18^O_2_ (Euriso‐top, Saint‐Aubin, France) with our IMAU working gas O_2_. The enriched O_2_ was then measured before and after passing through the GC column to quantify the isotopic reordering in the GC column and the source of the mass spectrometer.

### 2.6 Tropospheric and stratospheric air sampling and measurements

To determine the clumped isotope composition in lower tropospheric air O_2_, and to check the reproducibility of measurements, we have used ambient air compressed to ~140 bar in a ~40‐L cylinder. This air was collected outside the Center for Isotope Research on the campus of Groningen University (Groningen, The Netherlands), in August 2017. Air is compressed using a Rix oil‐free air and gas compressor (RIX Industries, Benicia, CA, USA). O_2_ is extracted from the compressed air cylinder as described in section 2.4 and the isotopic composition is measured following the procedure described in section 2.2. We also collected an air sample from the Utrecht University Campus (Utrecht, The Netherlands) on 18 September 2018 and measured its isotopic composition.

Stratospheric and upper tropospheric air samples were obtained with a whole air sampler operated on the high‐altitude aircraft M55 GEOPHYSICA on two flights carried out within the project StratoClim (stratospheric and upper tropospheric processes for better climate predictions). StratoClim is a collaborative research project under the European Commission's 7th Framework program (H2020) to improve the understanding of the key processes in the Upper Troposphere and Stratosphere with the goal of producing more reliable climate projections. We analyzed 13 samples collected on 1 and 6 September, 2016 during the StratoClim campaign over the Mediterranean (base airport Kalamata, Greece) at heights between 10 000 and 20 000 m and covering latitudes between 33 and 41°N and longitudes between 22 and 29°E. The temperature was −58°C near the highest Geophysica sampling altitudes (~ 20 km) and −42°C at the lowest sampling altitudes (~10 km). Stainless steel canisters of ~2 L volume were filled and compressed to ~4 bar with ambient air at different heights. O_2_ was extracted as described in section 2.4. Stable isotope ratios including Δ_35_ and Δ_36_ values were measured on purified O_2_ in the 253 Ultra as discussed in section 2.2.

The N_2_O mole fractions are measured together with the N_2_O isotopic composition in a Delta Plus XP continuous flow stable isotope ratio mass spectrometer (Thermo Fisher Scientific) following the method developed by Röckmann et al.^18^ Briefly, the air samples are passed through an Ascarite (sodium hydroxide coated silica; Sigma‐Aldrich, Inc., St Louis, MO, USA) column to remove CO_2_, and the N_2_O (together with remaining traces of CO_2_ and other condensable species) is preconcentrated cryogenically. The cryo‐focused sample is purified on a capillary GC column (PoraPlot Q, 0.32 mm; Agilent Technologies, GA, USA) and N_2_O is introduced into the mass spectrometer via an open split interface. From the area of the peaks, the concentration of N_2_O is calculated.

## 3 RESULTS AND DISCUSSION

### 3.1 Experimental results

#### 3.1.1 Zero enrichment and counting statistics

Table 1 presents the results from the zero‐enrichment measurements. The zero‐enrichment values vary in the ranges of −0.001 to 0.03‰ for δ^33^ values, −0.004 to 0.047‰ for δ^34^ values, −0.176 to 0.112‰ for δ^35^ values and 0.008 to 0.132‰ for δ^36^ values (Table 1). For the δ^33^ and δ^34^ values, the external errors of the six measurements are greater than the individual internal errors, which indicates that the mass spectrometer is not limiting the precision but an extra error of the order of 0.01‰ is introduced from other sources such as sample handling. For the clumped isotopes, the internal errors are larger and similar to the external errors of the six measurements, showing that the mass spectrometer precision (in principle the counting statistics) is the limiting factor here. A slightly positive value is observed for δ^36^, but it is within the error associated with the individual measurements. Therefore, a zero‐enrichment correction is not applied for the sample measurements.

**Table 1:** Results of zero‐enrichment measurements with bellows filled with IMAU‐O_2_. The errors are based on 30–60 cycles of sample and working gas measurements. Expected errors based on the counting statistics (EECS) are also presented. All δ values in this table are expressed in ‰ with respect to the working gas

Sl. No.	δ^33^ ± 1SE*	EECS	δ^34^ ± 1SE	EECS	δ^35^ ± 1SE	EECS	δ^36^ ± 1SE	EECS
1	0.030 ± 0.014	0.010	−0.006 ± 0.004	0.004	0.050 ± 0.180	0.224	0.068 ± 0.111	0.138
2	−0.018 ± 0.009	0.009	0.008 ± 0.004	0.004	−0.108 ± 0.167	0.223	0.087 ± 0.142	0.138
3	−0.001 ± 0.007	0.008	−0.004 ± 0.004	0.003	0.112 ± 0.143	0.177	0.039 ± 0.111	0.119
4	0.021 ± 0.014	0.013	0.004 ± 0.006	0.005	−0.176 ± 0.197	0.280	0.091 ± 0.187	0.189
5	−0.008 ± 0.009	0.009	−0.010 ± 0.004	0.004	−0.071 ± 0.165	0.198	0.008 ± 0.119	0.134
6	0.013 ± 0.010	0.010	0.047 ± 0.005	0.004	−0.157 ± 0.196	0.215	0.132 ± 0.097	0.138
Average ± 1σ	0.006 ± 0.018		0.006 ± 0.020		−0.058 ± 0.115		0.071 ± 0.043	

* 1 Standard Error.

The measured errors for all the isotopic ratios including the δ^35^ and δ^36^ values are compared with the errors expected from counting statistics and found to be similar (EECS, see Table 1 and supporting information). This proves that the 253 Ultra is very stable over the 7‐h duration of these measurements. The variation in the EECS is due to different signal strength and measurement duration. As the measurement uncertainty closely follows the counting statistics, the precision of measurements can be improved by increasing the signal intensity or the measurement time. For the present measurements we targeted an individual precision of 0.2 and 0.1‰ for Δ_35_ and Δ_36_, respectively. Higher precisions (e.g. Yeung et al^7^ reported an external reproducibility of 0.038‰) can probably be achieved by increasing measurement time and/or intensity.

We regularly observe when using the CDDs that the measurement errors are better than the counting statistics limit. We tentatively attribute this to the fact that the electron multipliers may not properly sample the Poisson distribution of count rates, because of the detector dead times. The Poisson distribution is the underlying statistics for calculating the counting statistics limit, and, if the high count rate tail is not detected, part of one tail of the distribution is cut off, which may lead to lower calculated errors for the CDDs. In the case of Faraday cups, all ions are counted as they pass through a resistor and the errors are similar to the counting statistics limit as expected.

#### 3.1.2 Ar and other isobaric interference

**Figure 3:**
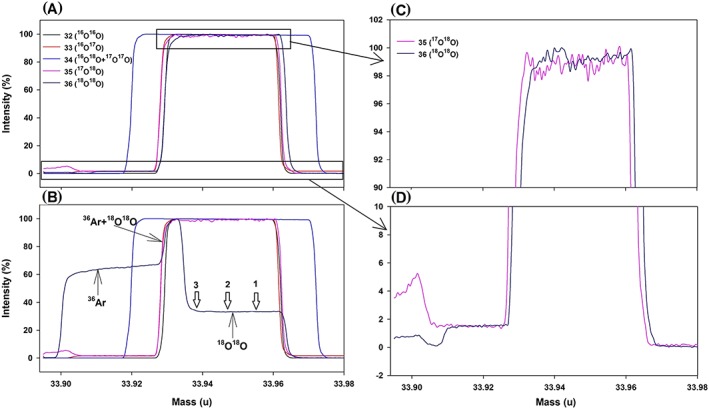
Peak scan for (A) pure O_2_ and (B) 0.16% Ar mixed with O_2_. All the five detector traces for measuring masses 32, 33, 34, 35 and 36 are shown. Although there is a partial peak overlap, the ^36^Ar peak does not interfere with the measurement of ^18^O^18^O when the measurement is made at the right half of the ^18^O^18^O peak (right side of position 2, indicated by arrows on the top of the ^18^O^18^O mass spectrum in B). Interference of ^36^Ar was observed when measured at position 3 (see text and Table 2). All the measurements for the air samples presented here were made at position 1. Magnified views of the top and bottom 10% of the peaks for masses 35 and 36 are shown in (C) and (D). The noise on the top (C) (~2%) could be reduced with longer integration. Significantly higher baselines for masses 35 and 36 on the left of the peaks (D) are due to contribution from ^35^Cl and H^35^Cl (see text). The small peak (~5% of the maximum) for mass 35 on the far left of the actual peak, observed occasionally, could be due to contamination but does not influence the ^17^O^18^O measurement [Color figure can be viewed at wileyonlinelibrary.com]

It is difficult to achieve full separation of Ar from O_2_ by passing the gases through a GC column. Some traces of Ar from the tail of the Ar peak always remain in the extracted O_2_ with the current GC separation setup. The influence of ^36^Ar (mass 35.9675 u) on ^18^O^18^O (mass 35.9983 u) is demonstrated using an Ar‐O_2_ mixture gas that is prepared by mixing 0.16% pure Ar with our pure O_2_ working gas. The mass difference between these two isobars is 0.0308 u and the resolving power required to separate their peaks is ~1160, which is easily achieved with the 253 Ultra at medium mass resolution. Figure 3 shows peak scans for pure O_2_ and the Ar‐O_2_ mixture. The O_2_ isotopologue peaks are about 0.03 u wide and they are in general very stable in terms of signal intensity, width of the plateau and collector positions. In the Ar‐O_2_ mixture, the ^36^Ar peak appears about 0.03 u before the ^18^O^18^O. There is a region of 0.009 u where the peaks overlap. The tilted peak for ^36^Ar is probably due to inaccurate adjustment of the CDD operating voltage. With further tuning of the operating voltage we later also achieved a flat peak for ^36^Ar (not shown here).

In addition to ^36^Ar, other interferences can be identified by the mass differences relative to the O_2_ isotopologue peaks. Chlorinated species are particularly important, especially in new mass spectrometers, as chlorinated solvents are used to degrease the parts, and we have regularly observed increases in these interferences after heating the ion source. ^35^Cl (mass 34.9688 u) causes the apparent increase in the background (~2% of the ^17^O^18^O peak height in Figure 3D) on the low‐mass side of the ^17^O^18^O peaks. It is detected 0.02 u before the ^17^O^18^O peak, and leaves the detector again at 0.01 u into the ^17^O^18^O peak. The effect of H^35^Cl (mass 35.9767 u) on ^18^O^18^O is visible on the left side of the ^18^O^18^O peak in the case of pure O_2_ (Figure 3D). However, for the Ar‐O_2_ mixture (Figure 3B), it is barely visible because it is superimposed on (and partly contributes to) the tilted slope of the ^36^Ar peak. Note that mass interferences conceptually cause “step changes” when they enter and exit the detector, and not tilted peak tops. The interferences from these isobars can easily be avoided in the 253 Ultra by measuring the isotopologue ratios at the right position of the peaks as the mass resolving power used for the present configuration (~10 000) is much higher than that required to resolve most of these isobars (e.g., 1664 for resolving ^18^O^18^O and H^35^Cl, and 1188 for resolving ^17^O^18^O and ^35^Cl). We note that the background for mass 33 is ~1.7% of the maximum peak height (Figures 3A and 3B). It increases about 0.02 u before the ^16^O^17^O peak but does not return to zero after the peak, as checked by extending mass scanning on the right side of the ^16^O^17^O peak. If it were due to contamination from interfering species (e.g., ^33^S and H^16^O^16^O), the background signal should return to zero after the width of an interfering peak of 0.03 u, as the width of any species falling on any cup other than the fixed Centre cup is ~0.03 u (Figure 3). We could not identify the cause of this offset, but it is similar for both sample and working gas and expected to have little effect on the final δ^33^ values.

Table 2 shows the isotopic ratios measured on pure O_2_ and the Ar‐O_2_ mixture at different positions of the peaks, as indicated in Figure 3B. A clear ^36^Ar interference to ^18^O^18^O is observed when measurements are made near the ^36^Ar peak, i.e., position 3 in Figure 3B and last line in Table 2. In addition, the internal error is highest for this measurement, indicating that small shifts in the mass scale change the contribution of ^36^Ar during the measurement period. Away from the ^36^Ar peak (positions 2 and 1), no significant ^36^Ar interference is observed (Table 2). We conclude that with the 253 Ultra, ^18^O^18^O can be easily resolved from ^36^Ar and other interferences even at medium resolution (resolving power: ~10 000 in this case). Therefore, interference‐free measurements of the clumped isotopic composition of O_2_ are possible even if the samples are not fully free from Ar. All the measurements presented here are made near the right edge of the peak plateauxs (position 1 in Figure 3B).

**Table 2:** Isotopic composition (δ^17^O and δ^18^O values in ‰ vs VSMOW) for the IMAU O_2_ working gas and a mixture of IMAU O_2_ and Ar to study the isobaric interference of ^36^Ar on the measurement of ^18^O^18^O. Measurements are made at different peak positions as shown in Figure 3. The ^36^Ar isobaric interference for Δ_36_ is very prominent when the measurement is made near the right edge of the Ar peak (position 3 in Figure 3)

Sample and peak positioning	δ^17^O (VSMOW)	δ^18^O (VSMOW)	Δ_35_ (ARF)	Δ_36_ (ARF)
Pure O_2_	9.260 ± 0.007	18.548 ± 0.008	1.262 ± 0.115	2.475 ± 0.043
O_2_–Ar mixture at peak position 1	9.173 ± 0.004	18.412 ± 0.008	1.236 ± 0.153	2.412 ± 0.086
O_2_–Ar mixture at peak position 2	9.178 ± 0.005	18.391 ± 0.006	1.190 ± 0.115	2.331 ± 0.101
O_2_–Ar mixture at peak position 3	9.174 ± 0.012	18.391 ± 0.008	1.303 ± 0.151	21.419 ± 0.257

The small but significant differences in δ^17^O and δ^18^O values between pure O_2_ and the Ar‐O_2_ mixture (Table 2) are attributed to isotope fractionation during sample handling in the gas mixing process. No significant differences are observed for Δ_35_ or Δ_36_ values, indicating that fractionation during sample handling, if any, is within the measurement uncertainty (discussed in sections 3.1.3 and 3.1.5).

#### 3.1.3 Isotopic reordering in the GC column and the source of the mass spectrometer

To test isotopic reordering in the GC column and in the source of the mass spectrometer, isotopically spiked O_2_ (Δ_36_ ~684‰ and Δ_35_ ~31‰) is used (Table 3). The spiked O_2_ sample is analyzed after preparation and then again after 4 days of storage in a stainless‐steel canister of the same type as used for the stratospheric air samples presented below. The results agree within the analytical errors, suggesting that isotopic reordering in this storage canister is insignificant (Table 3).

**Table 3:** Changes in the isotopic composition (δ and Δ values are given in ‰) of enriched O_2_ due to isotopic reordering in the GC column and storage in stainless‐steel canister

Sample	δ^33^ (sam‐wg)	δ^34^ (sam‐wg)	δ^35^ (sam‐wg)	δ^36^ (sam‐wg)	δ^17^O (VSMOW)	δ^18^O (VSMOW)	Δ_35_ (ARF)	Δ_36_ (ARF)	% change in Δ_36_
IMAU O_2_	0.006 ± 0.018	0.006 ± 0.020	−0.058 ± 0.115	0.071 ± 0.043	9.260 ± 0.007	18.548 ± 0.008	1.262 ± 0.115	2.475 ± 0.043	
Spiked O_2_	0.015 ± 0.010	0.030 ± 0.005	31.093 ± 0.150	700.107 ± 0.201	9.270 ± 0.010	18.573 ± 0.005	31.398 ± 0.115	684.252 ± 0.284	
Spiked O_2_ stored 4 days in a canister	0.006 ± 0.027	0.027 ± 0.019	30.476 ± 0.377	699.935 ± 0.368	9.260 ± 0.027	18.570 ± 0.020	30.808 ± 0.205	684.004 ± 0.228	0.04
GC separation at −75°C	0.066 ± 0.009	0.151 ± 0.003	31.258 ± 0.223	689.285 ± 0.0159	9.321 ± 0.010	18.696 ± 0.004	31.384 ± 0.321	673.297 ± 0.143	1.6
GC separation at −80°C	0.074 ± 0.020	0.168 ± 0.008	31.042 ± 0.453	688.600 ± 0.290	9.329 ± 0.021	18.713 ± 0.008	31.149 ± 0.377	672.569 ± 0.012	1.7

To investigate reordering in the purification system (in particular the GC column), an aliquot of the isotopically spiked O_2_ is mixed with pure helium to prepare an O_2_ concentration of ~20%, similar to atmospheric O_2_, and the mixture is passed through the purification system following the same procedure as for the samples. The measured isotope ratios before and after passing through the purification system are presented in Table 3. From the changes in Δ_35_ and Δ_36_ we conclude that the isotopic reordering in the purification system (GC column) is 1.6% when He‐O_2_ is passed through the column cooled to −75°C and 1.7% at −80°C. For our measurements of atmospheric O_2_ with a typical Δ_36_ value of 2.4‰, 1.7% isotopic reordering towards a scrambled isotopic distribution would induce a systematic error of ~0.04‰ (assuming the same relative reordering of 1.7%), which is below our reported uncertainty. The observed values for isotopic reordering are similar to the 1.1% reported by Yeung et al^11^ for their system.

The measurement of the spiked gas itself can also provide information about possible isotope reordering in the ion source of the mass spectrometer.^11^ Significant isotopic reordering of such a highly enriched gas due to fragmentation‐recombination reactions inside the ion source would change the δ^34^ value of the spiked O_2_ compared with the unspiked O_2_. This may occur because in the fragmentation‐recombination process, some ^18^O^18^O will be converted into ^18^O^16^O. The natural abundance of ^18^O^18^O is 4.2*10^−6^; therefore, the spiked gas with Δ_36_ ~684‰ contains 2*684‰*4.2*10^−6^ ≈ 5.7*10^−6^ extra ^18^O atoms_._ If 1% of the added ^18^O^18^O is converted into ^18^O^16^O, this would produce 5.7*10^−8 18^O^16^O molecules, which would cause a change in the δ^34^ value of 0.014‰. The actually observed δ^34^ difference between the spiked and unspiked O_2_ is 0.02‰ (Table 3) which is similar to the analytical error of 0.02‰ (Table 1). This indicates that the isotopic reordering in the source of the mass spectrometer is less than 2%. The spiked O_2_ has 97.2 atom % ^18^O^18^O. If the remaining 2.8% is ^18^O^16^O, this would change the ^18^O^16^O of the IMAU O_2_ by ~0.007‰. We conclude that isotopic reordering effects in storage canisters, in the purification system and in the source of the mass spectrometer are below our reported measurement errors and no corresponding corrections are applied.

#### 3.1.4 Calibration and reporting the clumped isotope ratios on the absolute reference frame

**Figure 4:**
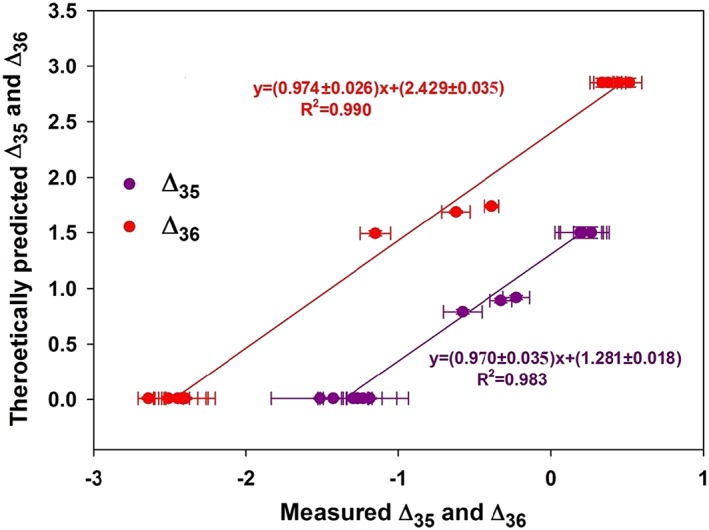
Construction of an empirical transfer function for conversion of measured Δ_35_ and Δ_36_ values (expressed against our working gas) into the absolute reference frame (ARF). Theoretical values (y axis) are those predicted by equilibrium thermodynamics.^13^ The equilibration of O_2_ induced by UV radiation is carried out at temperatures of −63, 4, 8 and 26°C (see text) and heated O_2_ is prepared at 850°C. The errors are 1 SE for 30–60 cycles of measurement. The errors in the theoretical values due to uncertainty in temperature estimates are discussed in the text [Color figure can be viewed at wileyonlinelibrary.com]

The Δ_35_ and Δ_36_ values of the O_2_ that is isotopically equilibrated at different temperatures (−63°C, 4°C, 8°C, 26°C and 850°C) are plotted against the corresponding values calculated for O_2_ in thermodynamic isotope equilibrium^12^, ^19^ in Figure 4 (the numerical values are provided in Table S1, supporting information). The linear fit to the data presented in Figure 4 provides an empirical transfer function to convert the measured Δ_35_ and Δ_36_ values for O_2_ into the Absolute Reference Frame (ARF).^15^ The transfer functions (weighted regression linear fit lines) for Δ_35_ and Δ_36_ in the present case are Δ_35(ARF)_ = (0.970 ± 0.035), Δ_35(meas)_ + (1.281 ± 0.018) and Δ_36(ARF)_ = (0.974 ± 0.026), Δ_36(meas)_ + (2.429 ± 0.035), respectively (Figure 4). The slopes of the best‐fit lines are close to 1, indicating close agreement between the theoretical prediction and measurements. This is additional evidence that isotopic reordering in the source of the mass spectrometer is insignificant, since isotopic reordering would lead to scale compression and result in slopes greater than 1 for the empirical transfer functions. The intercepts of the transfer functions indicate the Δ_35_ and Δ_36_ values (Δ_35_ = 1.281 and Δ_36_ = 2.429‰) of our working gas (IMAU‐O_2_).

For the samples analyzed, the Δ_35_ and Δ_36_ values before and after application of the transfer function are provided in Table S2 (supporting information). The measured Δ_35_ and Δ_36_ values before converting to the absolute scale are the measured values of the samples versus those of the heated gas assuming that the heated gas is isotopically scrambled. No significant difference is observed in the Δ_35_ and Δ_36_ values before and after conversion to the ARF (Table S2, supporting information). Our scientific interpretations below are based on the Δ_35_ and Δ_36_ values after conversion to the ARF, which facilitates inter‐laboratory comparison.

#### 3.1.5 Reproducibility and accuracy

We measured the isotopic composition including Δ_35_ and Δ_36_ values of O_2_ from a compressed air cylinder throughout the duration of sample measurements to monitor the reproducibility as well as possible long‐term drift or variability, if present. The results of the compressed air measurements are presented in Table 4. Errors in the δ^17^O and δ^18^O values were larger during the initial phase of measurements, possibly due to instability of the 253 Ultra, which caused some drifts in the mass scale and changes in signal sensitivity. With the passage of time, the δ^17^O and δ^18^O values became more stable and the measurement errors in the later phase are similar to the errors expected from counting statistics (Table 4). As for the zero enrichment (Table 1), the variations in the δ^17^O and δ^18^O values between measurements are larger than the measurement errors, which probably originate from fractionation in sample purification and handling. The values of Δ^17^O, Δ_35_ and Δ_36_ and their uncertainties are close to the counting statistics limit throughout the measurement period. The smaller variability in Δ^17^O, Δ_35_ and Δ_36_ is probably because the fractionations associated with handling are mass dependent and do not affect the second‐order isotope signatures. The long‐term (3 months) external reproducibility (1σ standard deviation) of the measured values is 0.115‰ for δ^17^O, 0.215‰ for δ^18^O, 0.021‰ for Δ^17^O, 0.101‰ for Δ_35_ and 0.098‰ for Δ_36_ (Table 4). Table 4 (bottom row) also shows results for the air sample collected at the Utrecht University Campus. The isotopic results are similar to those of the air from the reference compressed air cylinder.

**Table 4:** Isotopic composition including Δ_35_ and Δ_36_ values of tropospheric air in ‰ vs VSMOW (from reference compressed air cylinder and air from Utrecht University campus, The Netherlands) measured at different times during the measurement period. The measured standard errors and the errors expected based on the counting statistics (EECS) are also presented

Sl no	Date of measurement	δ^17^O (VSMOW)	EECS	δ^18^O (VSMOW)	EECS	Δ^17^O	EECS	Δ_35_ (ARF)	EECS	Δ_36_ (ARF)	EECS
1	19‐3‐18	11.893 ± 0.010	0.010	23.654 ± 0.014	0.003	−0.240 ± 0.012	0.010	1.391 ± 0.152	0.167	2.547 ± 0.090	0.103
2	19‐3‐18	11.965 ± 0.013	0.008	23.742 ± 0.019	0.003	−0.214 ± 0.016	0.008	1.447 ± 0.155	0.167	2.517 ± 0.097	0.104
3	21‐3‐18	11.977 ± 0.063	0.008	23.855 ± 0.035	0.003	−0.259 ± 0.064	0.008	1.414 ± 0.153	0.170	2.524 ± 0.092	0.106
4	03‐4‐18	12.182 ± 0.010	0.010	24.125 ± 0.008	0.004	−0.192 ± 0.010	0.010	1.213 ± 0.225	0.224	2.391 ± 0.146	0.139
5	04‐4‐18	12.088 ± 0.007	0.008	24.016 ± 0.004	0.004	−0.230 ± 0.007	0.008	1.212 ± 0.142	0.189	2.381 ± 0.101	0.120
6	04‐4‐18	12.146 ± 0.010	0.010	24.131 ± 0.007	0.005	−0.230 ± 0.010	0.010	1.360 ± 0.203	0.240	2.526 ± 0.178	0.156
7	04‐4‐18	11.912 ± 0.008	0.008	23.621 ± 0.004	0.004	−0.205 ± 0.008	0.008	1.136 ± 0.126	0.190	2.306 ± 0.093	0.119
8	12‐4‐18	11.912 ± 0.007	0.008	23.724 ± 0.003	0.004	−0.258 ± 0.007	0.008	1.289 ± 0.150	0.184	2.386 ± 0.094	0.120
9	24‐4‐18	11.958 ± 0.008	0.008	23.746 ± 0.008	0.003	−0.223 ± 0.009	0.008	1.281 ± 0.119	0.156	2.320 ± 0.088	0.101
10	02‐5‐18	11.832 ± 0.008	0.008	23.499 ± 0.004	0.003	−0.223 ± 0.008	0.008	1.360 ± 0.155	0.177	2.564 ± 0.102	0.110
Average ± 1σ		11.986 ± 0.115		23.811 ± 0.216		−0.227 ± 0.021		1.310 ± 0.101		2.447 ± 0.098	
Tropospheric air from Utrecht, The Netherlands											
1	18–9‐18	11.979 ± 0.013	0.014	23.752 ± 0.006	0.006	−0.205 ± 0.013	0.014	1.279 ± 0.248	0.298	2.356 ± 0.164	0.186

#### 3.1.6 Dependence of clumped isotope ratio (Δ) on bulk isotope ratio (δ value)

Clumped isotope measurements with isotope ratio mass spectrometers may exhibit a dependence of the clumped isotope ratio Δ on the bulk isotopic signature δ. A corresponding correction is found to be necessary for most of the CO_2_ clumped isotope measurements using low‐resolution isotope ratio mass spectrometers.^15^, ^19^, ^20^, ^21^ In order to examine whether such an effect is present for our clumped isotope measurements of O_2_ with the 253 Ultra, we isotopically scrambled two gases with widely different bulk isotopic compositions at high temperature (850°C). The isotopic ratios of the two gases, viz. IMAU O_2_ and GEO O_2_ before and after heating, are presented in Table 5. IMAU O_2_ is our working gas and GEO O_2_ is a commercial O_2_ gas supplied by Air Liquide (Eindhoven, The Netherlands). GEO O_2_ has very low bulk isotope ratios and high Δ_35_ and Δ_36_ values compared with IMAU‐O_2_, but after isotopic equilibration at 850°C (for 2–3 h) in the presence of platinum, the Δ_35_ and Δ_36_ values of the two O_2_ gases are identical, indicating full isotopic scrambling at this temperature. This also indicates the absence of any dependence of the Δ_35_ and Δ_36_ values on the bulk isotopic composition within our measurement precision. We note that heating does affect the δ^17^O and δ^18^O values, which is possibly due to some exchange with oxygen in the quartz material of the reactor at high temperature. However, this exchange does not affect the Δ_35_ and Δ_36_ values as these signatures are purely driven by exchange temperatures.

**Table 5:** Stable isotopic composition (in ‰) including Δ_35_ and Δ_36_ values of the laboratory standards (IMAU O_2_ and GEO O_2_) before and after heating. Heating is carried out at 850°C for more than 2 h in presence of platinum sponge. The δ^35^ and δ^35^ values are expressed against IMAU O_2_

O_2_ gas	δ^17^O (VSMOW)	δ^18^O (VSMOW)	δ^35^ (sam‐wg)	δ^36^ (sam‐wg)	Δ_35_ (ARF)	Δ_36_ (ARF)
Before heating						
IMAU O_2_	9.260 ± 0.007	18.548 ± 0.008	−0.060 ± 0.049	0.068 ± 0.020	1.262 ± 0.115	2.475 ± 0.043
GEO O_2_	−20.729 ± 0.002	−37.921 ± 0.018	−81.962 ± 0.179	−105.984 ± 0.104	2.821 ± 0.128	4.423 ± 0.114
After heating at 850°C						
IMAU O_2_	10.919 ± 0.014	21.899 ± 0.004	3.671 ± 0.109	4.178 ± 0.146	0.005 ± 0.116	0.027 ± 0.085
GEO O_2_	−18.549 ± 0.137	−33.647 ± 0.226	−78.327 ± 0.243	−102.022 ± 0.413	0.108 ± 0.105	0.080 ± 0.080

#### 3.1.7 Possibility of measuring ^17^O^17^O in the 253 Ultra

We also investigated the possibility of measuring the rarest isotopologue ^17^O^17^O (mass 33.99826; 0.032 ppm in the atmosphere). The biggest challenge is the interference from ^16^O^18^O (mass 33.99408; abundance 4100 ppm). The required mass resolution to separate these two masses is 8132, so they can in principle be resolved, in the medium‐ or high‐resolution mode. However, the detection is complicated by the very low abundance of ^17^O^17^O compared with^16^O^18^O. Under the present measurement conditions (at medium resolution) the expected signal strength for ^17^O^17^O is 1450 cps when the signal for its isobar ^16^O^18^O is 4.11*10^7^ cps, so ^17^O^17^O needs to be resolved on the tail of the very large ^16^O^18^O peak. When going to high‐resolution mode the signals decrease correspondingly, and the small signal of ^17^O^17^O (a few hundred cps) is only observable with a CDD. High‐precision determination of ^17^O^17^O will therefore take even longer than that of the more abundant clumped isotopes. The ^17^O^17^O isotopologue is expected to carry similar information to the other two clumped isotopologues and its measurement is therefore not yet being targeted rigorously.

### 3.2 Stratospheric and tropospheric clumped isotope ratios

The Δ_35_ and Δ_36_ values along with the conventional isotope ratios measured in the stratospheric and upper tropospheric air O_2_ are presented in Table 6. The δ^17^O and δ^18^O values show only a small spread, between 11.89 and 11.98‰ for δ^17^O values and between 23.60 and 23.77‰ for δ^18^O values (Table 6), and are indistinguishable from those observed in the lower troposphere (Table 4). The δ^17^O and δ^18^O values are in agreement with numerous previous investigations that span the range of 11.98 to 12.26‰ for δ^17^O values and 23.53 to 24.15‰ for δ^18^O values.^22^, ^23^, ^24^ The average ^17^O anomaly, defined by Δ^17^O = ln(δ^17^O + 1) − 0.516*ln(δ^18^O + 1) is found to be −0.23 ± 0.03‰. Note that the three‐isotope slope (λ) value used here is 0.516. When using the same slope, the data from Luz and Barkan^25^ yield a Δ^17^O value of −0.17, close to our result. Laskar et al^26^ reported the Δ^17^O value of atmospheric O_2_ to be −0.22 ± 0.02‰ based on indirect measurement of car exhaust CO_2_, as the source of the O_2_ in the exhaust's CO_2_ is atmospheric O_2_, consumed during the combustion of fuel. The agreement with previous values for the bulk isotopes including Δ^17^O provides confidence that the extraction of O_2_ from air is made without strong fractionation.

**Table 6:** Sampling information and stable isotopic composition (in ‰) including Δ_35_ and Δ_36_ values of stratospheric and upper tropospheric O_2_ samples collected on the GEOPHYSICA M55 aircraft over the Mediterranean. The samples were collected on 1 and 6 September 2016

Alt (km)*	Latitude (^o^N)#	Longitude (°E) #	Temp (°C)†	δ^17^O (VSMOW)	δ^18^O (VSMOW)	Δ^17^O	Δ_35_ (ARF)	Δ_36_ (ARF)
20.0–20.2	35.52	25.13	−58.7	11.920 ± 0.007	23.681 ± 0.003	−0.227 ± 0.007	1.439 ± 0.132	3.078 ± 0.073
20.0	34.52	27.79	−59.5	11.947 ± 0.006	23.711 ± 0.003	−0.216 ± 0.006	1.630 ± 0.129	2.950 ± 0.120
16.5–20.0	40.34	24.95	−61.1	11.918 ± 0.008	23.634 ± 0.003	−0.206 ± 0.008	1.579 ± 0.086	2.978 ± 0.088
20.0	39.06	25.57	−59.8	11.930 ± 0.009	23.736 ± 0.003	−0.245 ± 0.009	1.572 ± 0.093	3.021 ± 0.086
19.5–19.6	33.60	30.42	−64.1	11.975 ± 0.008	23.709 ± 0.003	−0.187 ± 0.008	1.657 ± 0.103	3.097 ± 0.121
19.4–19.7	36.70	27.07	−63.3	11.920 ± 0.007	23.603 ± 0.004	−0.188 ± 0.007	1.579 ± 0.088	2.903 ± 0.044
18.4–19.1	34.54	25.68	−66.4	11.892 ± 0.008	23.679 ± 0.003	−0.254 ± 0.008	1.653 ± 0.082	2.914 ± 0.073
17.6	34.30	28.57	−67.4	11.941 ± 0.007	23.652 ± 0.003	−0.192 ± 0.007	1.551 ± 0.118	2.865 ± 0.051
17.5–17.6	35.11	26.12	−63.3	11.945 ± 0.007	23.662 ± 0.003	−0.193 ± 0.007	1.605 ± 0.093	2.889 ± 0.060
17.5	35.58	24.89	−63.4	11.901 ± 0.008	23.770 ± 0.003	−0.291 ± 0.008	1.535 ± 0.163	2.923 ± 0.089
14.8–15.4	36.56	22.58	−60.8	11.951 ± 0.007	23.640 ± 0.003	−0.176 ± 0.007	1.092 ± 0.143	2.436 ± 0.092
9.7–10.8	36.24	23.18	−42.5	11.912 ± 0.008	23.670 ± 0.004	−0.229 ± 0.008	1.301 ± 0.156	2.518 ± 0.110
10.7–11.2	40.91	23.67	−48.7	11.906 ± 0.007	23.680 ± 0.003	−0.241 ± 0.007	1.137 ± 0.147	2.225 ± 0.077

* The air samples were collected during a period of ~ 5 min, the range of the altitude covered during sampling is given.

# Latitude and longitude at the starting point of sampling.

† Temperature is the average temperature over the collection duration.

**Figure 5:**
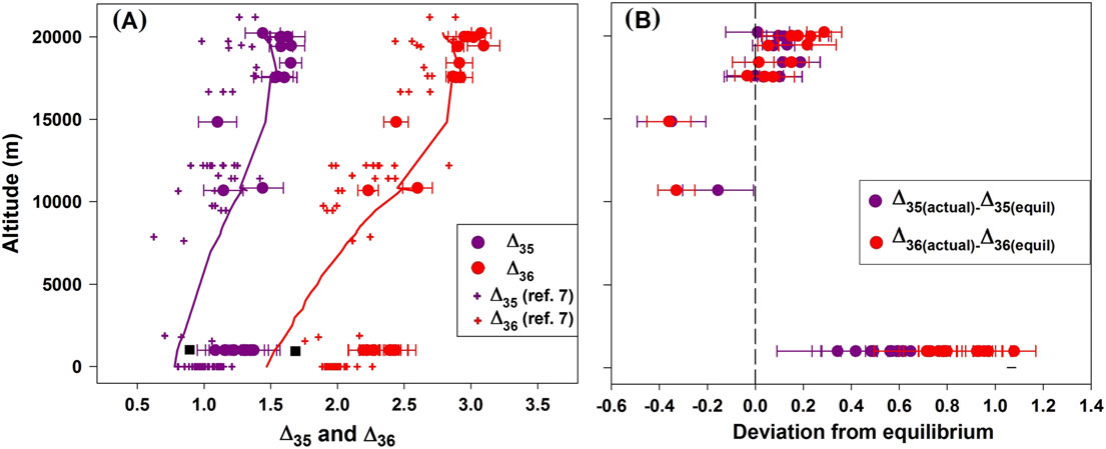
(A) Vertical profiles of Δ_35_ and Δ_36_ for the StratoClim (GEOPHYSICA) samples. The Δ_35_ and Δ_36_ values plotted at 1000 m altitude represent multiple measurements of the tropospheric reference air cylinder that was filled in August 2017 at Groningen, The Netherlands, and a sample from Utrecht, The Netherlands. They are plotted at an altitude of 1000 m to distinguish them from the data of Yeung et al^7^; the real altitude is ~0 m a.s.l. The solid lines are the equilibrium Δ_35_ and Δ_36_ values calculated for the ambient temperatures measured on the GEOPHYSICA aircraft. The black squares represent the equilibrium Δ_35_ and Δ_36_ values, estimated based on the local temperatures in The Netherlands during sampling. The Δ_35_ and Δ_36_ values in the stratosphere and troposphere reported by Yeung et al^7^ are also presented for comparison (in the altitude range covered by our data). Error bars indicate 1 SE for 30–60 cycles of measurement for each sample. (B) Deviation of the measured Δ_35_ and Δ_36_ values from the equilibrium values expected at the ambient temperatures [Color figure can be viewed at wileyonlinelibrary.com]

Figure 5 shows the Δ_35_ and Δ_36_ values measured in stratospheric and upper tropospheric O_2_ obtained from the GEOPHYSICA air samples. The values lie in the range of 1.1 to 1.6‰ for Δ_35_ and 2.2 to 3.1‰ for Δ_36_ (Table 6). The Δ_35_ and Δ_36_ values are higher in the stratospheric samples than in those from the troposphere. The Δ_35_ and Δ_36_ values of the lower tropospheric air O_2_ from the compressed air cylinder filled at Groningen and an air sample collected at the Utrecht University campus are also plotted in Figure 5 for comparison. The equilibrium Δ_35_ and Δ_36_ values calculated for the temperatures measured on the GEOPHYSICA aircraft during the sampling flights are plotted for comparison (lines in Figure 5A). The equilibrium Δ_35_ and Δ_36_ values at the time of sampling at Groningen are shown by the black square. Figure 5B shows the difference between the measured and the calculated equilibrium values. Stratospheric O_2_ is close to thermodynamic equilibrium at the ambient temperatures, as the measured values closely follow the equilibrium line. Previous data^7^ showed that the rapid isotopic exchange due to the O(^3^ *P*) + O_2_ reaction in the stratosphere caused full thermodynamic equilibration above ~22 km, whereas equilibration was not complete below 22 km. For our samples in the lower stratosphere, due to larger errors, it is not possible to decide from the clumped isotope values alone whether the isotopic equilibration is complete. However, the correlation with N_2_O (see below) indicates that the air in the lower stratosphere still has a partially tropospheric character. This demonstrates that full isotope equilibrium has not yet been achieved in the lower stratosphere.

The upper tropospheric Δ_35_ and Δ_36_ values are lower than the equilibrium values, whereas the lower tropospheric values are higher (Figure 5B). This is due to the lower temperatures in the upper troposphere which correspond to higher Δ_35_ and Δ_36_ values predicted from thermodynamic equilibrium than in the lower troposphere. No significant difference in the Δ_35_ and Δ_36_ values between the upper and lower troposphere is observed, indicating that tropospheric O_2_ is vertically well mixed in terms of the clumped isotopic composition. In other words, the O_2_ isotopic reordering time in the troposphere is longer than typical mixing time scales. The isotopic reordering times have been estimated to be a year in the upper troposphere and several years near the surface using O atom distributions calculated from the GEOS‐Chem atmospheric chemistry model.^7^

The Δ_35_ and Δ_36_ values of stratospheric and tropospheric air O_2_ reported by Yeung et al^7^ up to the altitude of ~22 km are also presented in Figure 5A for comparison. In general, a clear distinction between the stratospheric and tropospheric Δ_35_ and Δ_36_ values is observed in both data sets. However, absolute differences of ~0.3 in Δ_35_ and ~0.4‰ in Δ_36_ between the two data sets are evident, which are beyond the analytical uncertainty of ~0.1‰ for both Δ_35_ and Δ_36_ (Table 4). The tropospheric Δ_35_ values measured by Yeung et al^7^ vary between 0.6 and 1.2‰ with an average of 1.0‰ and the Δ_36_ values between 1.7 and 2.3‰ with an average of ~2.0‰. The present values of tropospheric Δ_35_ vary between 1.1 and 1.4‰ with an average of 1.3‰ and of Δ_36_ between 2.3 and 2.6‰ with an average of ~2.4‰.

The two sets of samples have different geographical origins, but a difference of ~0.4‰ is not expected as the mixing times in the troposphere are fast (weeks within one hemisphere and 1 year for inter‐hemispheric exchange) compared with the isotope reordering time scale of the order of a decade. In addition, the samples from Yeung et al^7^ cover a wide range in time and space. We suggest that the differences are due to differences in isotope scales between the two laboratories, in particular the assignment of effective temperatures for exchange experiments which is used for calibration. In our laboratory we use the change in pressure as an indication of the effective gas temperature (see section 2.3), whereas Yeung et al^7^ used the surface‐weighted radiative power (Stefan‐Boltzmann law) of different cold and warm parts of the reactor to estimate the gas temperature. If we use the same approach for our experiments, the effective temperature for the lowest temperature experiment (cold bath at −77.8°C and Suprasil finger at +10°C) would be −55°C inside the chamber for this experiment, compared with −63 K for the pressure‐based approach. This could explain differences of 0.1‰ and 0.2‰ in the Δ_35_ and Δ_36_ values, respectively for the coldest reactor temperatures (which are similar to stratospheric temperatures). The discrepancy in estimating the effective temperature of equilibration of O_2_ inside the chamber is probably a major reason for the differences in the Δ_35_ and Δ_36_ values between the present measurements and those reported by Yeung et al.^7^ The effect on the tropospheric Δ_35_ and Δ_36_ values, however, would be smaller. Another potential point of concern is that the effective temperature, however determined, is an average over very different temperature conditions in the reactor, changing from hot near the illuminated Suprasil finger to cold at the outer surface. Thus, reactor geometry and illumination conditions may also play a role. Although the differences between stratospheric and tropospheric results reported by Yeung et al^7^ and our new results are similar and lead to similar conclusions, the significant discrepancy of the absolute values highlights the need for an inter‐laboratory comparison and careful assessment of the calibration scale.

**Figure 6:**
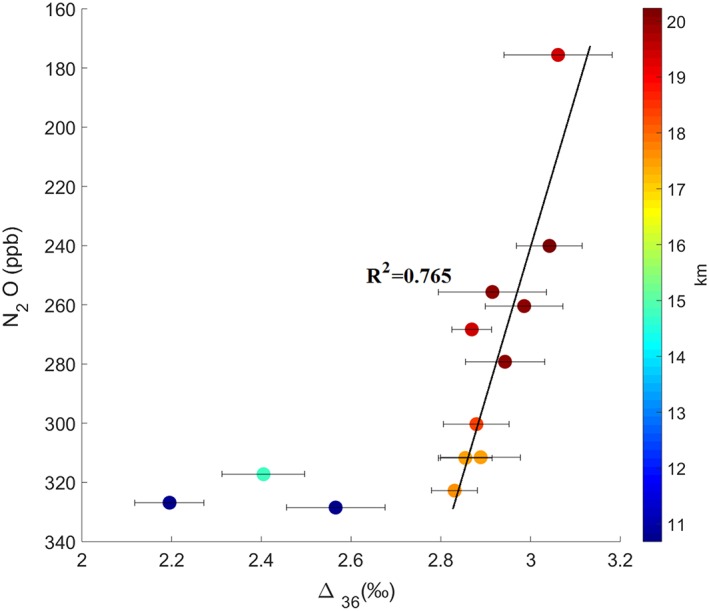
Cross plot of Δ_36_ and N_2_O. Color codes (scale on the right) indicate altitude at which samples were collected. The best‐fit line is drawn for the stratospheric samples only. Errors in N_2_O mole fractions are smaller than the symbol sizes [Color figure can be viewed at wileyonlinelibrary.com]

Figure 6 shows the correlation between Δ_36_ in O_2_ and the N_2_O mole fractions measured on the same samples. N_2_O is almost exclusively produced at the Earth's surface from natural and anthropogenic sources and destroyed almost completely in the stratosphere by UV photolysis and reaction with O(^1^ *D*).^27^, ^28^ Therefore, N_2_O is a useful tracer for stratospheric photochemistry and is often used as “pseudo vertical coordinate” for stratospheric studies, where lower N_2_O values indicate higher altitudes. A negative correlation is observed between Δ_36_ and N_2_O values for stratospheric samples (note that the N_2_O scale is reversed). The photochemical N_2_O removal is a slow process with timescales of months to years. The correlation between N_2_O and Δ_36_ therefore probably reflects mixing between fresh tropospheric air with higher N_2_O and lower Δ_36_ values and older stratospheric air with lower N_2_O and higher Δ_36_ values. This also implies that the lower stratospheric air is not in full isotopic equilibrium and retains some signatures of the troposphere, since isotope equilibration would eradicate the tropospheric signal and the correlation with N_2_O. The correlation is not observed in the troposphere because the troposphere is well mixed without any significant vertical gradient in N_2_O and Δ_36_.

## 4 CONCLUSIONS

We demonstrated that the 253 Ultra high‐resolution stable isotope ratio mass spectrometer allows clumped isotope analysis of O_2_ in atmospheric air. Isobaric interferences of ^36^Ar and H^35^Cl for ^18^O^18^O and ^35^Cl for ^17^O^18^O are easily resolved at medium mass resolution. Isotopic equilibration experiments using O_2_ heated to 850°C and photochemical isotope exchange via O(^3^ *P*) + O_2_ at lower temperatures are used to convert the measured values into the Absolute Reference Frame for clumped isotope analysis through an empirical transfer function. A critical parameter for this calibration is the effective isotope exchange temperature in the photolysis reactor, and uncertainties in assigning the correct temperature could be the cause of an absolute difference of ~0.4‰ between the data presented here and a previously published dataset.^7^ Analyzed stratospheric air samples collected with the M55 GEOPHYSICA aircraft show that the clumped isotopic composition of O_2_ is close to thermodynamic equilibrium at the ambient temperature, because of rapid equilibration due to the O(^3^ *P*) + O_2_ isotopic exchange reaction. However, the correlation with N_2_O suggests that isotopic equilibration is not complete in the lower stratosphere. The clumped isotope composition of tropospheric O_2_ deviates significantly from the thermodynamic equilibrium at the respective local temperatures and there is no significant vertical clumped isotope gradient in the troposphere. This shows that the isotopic resetting time (several years)^7^ is much smaller than the tropospheric vertical mixing time scale (weeks). These observations indicate that either isotopic reordering is only partial in the troposphere and tropospheric O_2_ retains part of the isotopic signature acquired in the stratosphere or that tropospheric O_2_ is isotopically reordered in the upper troposphere at an effective temperature of ~ −40°C. This is being investigated with measurements and model data.

## Supporting information

Table S1. Δ_35_ and Δ_36_ values of the equilibrated and heated O_2_ samples at different temperatures expressed in ‰ against IMAU O_2_, the working gas. These values are plotted against the corresponding thermodynamically predicted values to construct the empirical transfer functions (Figure 4 in the main text).
Table S2. Δ_35_ and Δ_36_ values before (measured) and after conversion to the absolute scale using the empirical transfer function (See Figure 4 in the main text). The measured clumped isotope data are the difference between the Δ_35_ and Δ_36_ values of the samples and the heated O_2_. Tropospheric air from the surface level was collected from Groningen and Utrecht, The Netherlands and stratospheric and upper tropospheric O_2_ samples were collected using the GEOPHYSICA M55 aircraft (see main text for details and discussion).

Data S1. Supporting information
