# Supplementary material for: Measurement of 18O18O and 17O18O in atmospheric O2 using the 253 Ultra mass spectrometer and applications to stratospheric and tropospheric air samples
Source: Rapid Commun Mass Spectrom. 2019 May 8;33(11):981–94. doi: 10.1002/rcm.8434 (PMC6594091; doi:10.1002/rcm.8434)
Supplement: Supplementary file 1 — Table S1. Δ35 and Δ36 values of the equilibrated and heated O2 samples at different temperatures expressed in ‰ against IMAU O2, the working gas. These values are plotted against the corresponding thermodynamically predicted values to construct the empirical transfer functions (Figure 4 in the main text). Table S2. Δ35 and Δ36 values before (measured) and after conversion to the absolute scale using the empirical transfer function (See Figure 4 in the main text). The measured clumped isotope data are the difference between the Δ35 and Δ36 values of the samples and the heated O2. Tropospheric air from the surface level was collected from Groningen and Utrecht, The Netherlands and stratospheric and upper tropospheric O2 samples were collected using the GEOPHYSICA M55 aircraft (see main text for details and discussion). [file RCM-33-981-s001.docx]

**Supporting information**

**Measurement of ^18^O^18^O and ^17^O^18^O in atmospheric O_2_ using the 253 Ultra mass spectrometer and applications to stratospheric and tropospheric air samples**

Amzad H. Laskar*, Rahul Peethambaran, Getachew A. Adnew and Thomas Röckmann

Institute for Marine and Atmospheric Research Utrecht, Utrecht University, The Netherlands

*Corresponding author: Phone +31302532924, e-mail: a.h.laskar@uu.nl

**S1. Measurement protocol and calculations of conventional and clumped isotopes**

The mass spectrometer bellows are evacuated and filled with the sample and the working gas (WG). The WG is IMAU O_2_. The signals are manually adjusted to match the target intensity in order to speed up the automatic signal balancing by the mass spectrometer. The instrument background is registered before each set of 10 acquisitions, with the dual inlet valve closed, for 60 s. This background is used by the Qtegra software to report background-corrected values. A peak center is performed on mass 32. A pressure adjust is performed to reach a target intensity of 7*10^9^-10*10^9^ cps on mass 32, with a tolerance of 0.3%. Then. six WG-sample measurement cycles are performed with an integration time of 67 s and an equilibration time of 60 s. From the registered signals the Qtegra software reports the background-corrected ion signals in counts per second (cps) for the 5 different detectors. Detailed calculations for one sample (sample # 9 in Table 4) are provided in the supporting Excel file.

In the supporting Excel file, columns A to H are directly obtained from the mass spectrometer. Parameters in the Excel file columns and the calculations are explained below:

A: Acquisition number

B: Bellow identifier

C: Serial number in an acquisition

D to H: Signal intensity in counts per second (cps) for mass 32 to 36

I to R: Signal intensity in cps for sample and average signal intensity of the two measurements of the working gas before and after the sample measurement

S to AB: Molecular isotopologue ratios e.g., 33/32, 34/32 for working gas and sample

AC, AD: Atomic isotope ratios ^17^O/^16^O (^17^R) and ^18^O/^16^O (^18^R) derived from the isotopologue ratios

AE to AH: δ^33^ to δ^36^ values expressed with respect to the working gas

AI, AJ: δ^17^O and δ^18^O values of the sample with respect to the working gas

AK: Difference in the signal intensity between the sample and the working gas. This is used for linearity corrections when the signals from the two bellows do not match and a dependence of the δ values on pressure is observed, referred to as pressure correction (pc)

AL to AQ: Pressure corrected δ_pc values. The pressure corrections are performed using the slopes of the linear fits to the correlation between δ values and the signal imbalance; the calculations are given in columns CB to DA

AT to BI: Average values, error estimates from measurements and counting statistics, clumped isotope calculations (detailed clumped isotope calculations in columns BO to BY), conversion of the measured conventional δ values to the VSMOW scale (cell# BD8 and BE8) and clumped isotope values to Absolute Reference Frame (Cell # BH13 and BI13).

**S2. Error calculation**

The reported standard error of the mean (*SE*) of the measurement is calculated using the relation $SE=\frac{1}{\sqrt{N}}\sqrt{\frac{\Sigma(x_{i}-\mu)^{2}}{N}}$, where $x_{i}$are the individual δ values, $\mu$is the mean of all measured δ values and *N* is the number of measurements. The error based on counting statistics is calculated based on Poisson’s distribution as discussed in Brand^1^. The expected error based on counting statistics (*EECS*) is calculated as $EECS=\sqrt{\frac{2}{cps\times t_{\mathrm{int}}\times N}}$, where *cps* is the average intensity in counts per second for a sample or working gas, *t_int_* is the integration time in seconds for each cycle and N is the number of cycles. The factor 2 takes into account that the measurements of sample and working gas each contribute the same error to the δ value.

Table S1. Δ_35_ and Δ_36_ values of the equilibrated and heated O_2_ samples at different temperatures expressed in ‰ against IMAU O_2_, the working gas. These values are plotted against the corresponding thermodynamically predicted values to construct the empirical transfer functions (Figure 4 in the main text).

| Temperature | Δ_35_ (sam-ref) | Δ_36_ (sam-ref) |
| --- | --- | --- |
| 850±10 ^o^C | -1.266±0.073 | -2.510±0.097 |
| 850±10 ^o^C | -1.430±0.086 | -2.449±0.077 |
| 850±10 ^o^C | -1.516±0.317 | -2.641±0.067 |
| 850±10 ^o^C | -1.301±0.194 | -2.428±0.110 |
| 850±10 ^o^C | -1.232±0.298 | -2.400±0.196 |
| 850±10 ^o^C | -1.186±0.177 | -2.423±0.173 |
| 850±10 ^o^C | -1.274±0.102 | -2.408±0.145 |
| 26±5 ^o^C | -0.580±0.126 | -1.150±0.100 |
| 8±5 ^o^C | -0.328±0.072 | -0.624±0.093 |
| 4±5 ^o^C | -0.227±0.092 | -0.389±0.042 |
| -63±5 ^o^C | 0.195±0.169 | 0.372±0.093 |
| -63±5 ^o^C | 0.202±0.140 | 0.448±0.044 |
| -63±5 ^o^C | 0.263±0.117 | 0.516±0.117 |
| -63±5 ^o^C | 0.191±0.140 | 0.334±0.080 |

Table S2. Δ_35_ and Δ_36_ values before (measured) and after conversion to the absolute scale using the empirical transfer function (See Figure 4 in the main text). The measured clumped isotope data are the difference between the Δ_35_ and Δ_36_ values of the samples and the heated O_2_. Tropospheric air from the surface level was collected from Groningen and Utrecht, The Netherlands and stratospheric and upper tropospheric O_2_ samples were collected using the GEOPHYSICA M55 aircraft (see main text for details and discussion).

| Measured data | | Converted to absolute scale | |
| --- | --- | --- | --- |
| Δ_35_ | Δ_36_ | Δ_35_ | Δ_36_ |
| Tropospheric air from Groningen, Netherlands | | | |
| 1.410 | 2.560 | 1.391 | 2.547 |
| 1.468 | 2.529 | 1.447 | 2.517 |
| 1.434 | 2.537 | 1.414 | 2.524 |
| 1.227 | 2.400 | 1.213 | 2.391 |
| 1.226 | 2.390 | 1.212 | 2.381 |
| 1.378 | 2.539 | 1.360 | 2.526 |
| 1.147 | 2.313 | 1.136 | 2.306 |
| 1.305 | 2.395 | 1.289 | 2.386 |
| 1.298 | 2.347 | 1.282 | 2.324 |
| 1.378 | 2.578 | 1.360 | 2.564 |
| Tropospheric air from Utrecht University Campus, Netherlands | | | |
| 1.295 | 2.364 | 1.279 | 2.356 |
| Stratospheric and upper tropospheric O_2_ samples from GEOPHYSICA M55 aircraft | | | |
| 1.460 | 3.105 | 1.439 | 3.078 |
| 1.657 | 2.974 | 1.630 | 2.950 |
| 1.604 | 2.926 | 1.579 | 2.978 |
| 1.597 | 3.047 | 1.572 | 3.021 |
| 1.684 | 3.125 | 1.657 | 3.097 |
| 1.604 | 2.926 | 1.579 | 2.903 |
| 1.681 | 2.937 | 1.653 | 2.914 |
| 1.576 | 2.886 | 1.551 | 2.865 |
| 1.631 | 2.911 | 1.605 | 2.889 |
| 1.559 | 2.947 | 1.535 | 2.923 |
| 1.103 | 2.446 | 1.092 | 2.436 |
| 1.289 | 2.500 | 1.301 | 2.518 |
| 1.149 | 2.230 | 1.137 | 2.225 |

**Reference**

1. Brand WA, Mass Spectrometer Hardware for Analyzing Stable Isotope Ratios, In de Groot PA, ed. *Handbook of Stable Isotope Analytical Techniques, Volume-I*. Elsevier Science, Amsterdam, 2004:835-858.
